# Organic mulching positively regulates the soil microbial communities and ecosystem functions in tea plantation

**DOI:** 10.1186/s12866-020-01794-8

**Published:** 2020-04-29

**Authors:** Shuning Zhang, Yu Wang, Litao Sun, Chen Qiu, Yiqian Ding, Honglian Gu, Linjun Wang, Zhaoshun Wang, Zhaotang Ding

**Affiliations:** 1 Tea Research Institute, Qingdao Agricultural University, Qingdao, 266109 Shandong China; 2 Weihai agricultural and rural affairs service center, Weihai, 264200 Shandong China

**Keywords:** *Camellia sinensis* (L.) O. Kuntze, Mulch, Bacterial and fungal communities, Tea plantation soil

## Abstract

**Background:**

Different mulches have variable effects on soil physicochemical characteristics, bacterial and fungal communities and ecosystem functions. However, the information about soil microbial diversity, community structure and ecosystem function in tea plantation under different mulching patterns was limited. In this study, we investigated bacterial and fungal communities of tea plantation soils under polyethylene film and peanut hull mulching using high-throughput 16S rRNA and ITS rDNA gene Illumina sequencing.

**Results:**

The results showed that the dominant bacterial phyla were Proteobacteria*,* Actinobacteria, Acidobacteria and Chloroflexi, and the dominant fungal phyla were Ascomycota, Mortierellomycota and Basidiomycota in all samples, but different mulching patterns affected the distribution of microbial communities. At the phylum level, the relative abundance of Nitrospirae in peanut hull mulching soils (3.24%) was significantly higher than that in polyethylene film mulching soils (1.21%) in bacterial communities, and the relative abundances of Mortierellomycota and Basidiomycota in peanut hull mulching soils (33.72, 21.93%) was significantly higher than that in polyethylene film mulching soils (14.88, 6.53%) in fungal communities. Peanut hull mulching increased the diversity of fungal communities in 0–20 cm soils and the diversity of bacterial communities in 20–40 cm soils. At the microbial functional level, there was an enrichment of bacterial functional features, including amino acid transport and metabolism and energy production and conversion, and there was an enrichment of fungal functional features, including undefined saprotrophs, plant pathogens and soils aprotrophs.

**Conclusions:**

Unique distributions of bacterial and fungal communities were observed in soils under organic mulching. Thus, we believe that the organic mulching has a positive regulatory effect on the soil bacterial and fungal communities and ecosystem functions, and so, is more suitable for tea plantation.

## Background

Mulching is an important measure in agricultural production, which is mainly used for soil improvement and environmental protection. According to the type of materials, it could be categorized as inorganic and organic mulching. Inorganic mulching was widely used as a low-cost and water-saving measure in agriculture areas that were susceptible to drought. Although it could improve soil moisture, prevent soil nutrient loss and control crop pests and diseases [1, 2], it could change the soil biological characteristics and negatively impact on soil quality and sustainability, and even cause soil alkalization, resulting in injuries to plants [3]. However, organic mulching is different, it is mainly derived from plant residues, which are proven to be better for soil health. The application of organic mulch on soils could not only inhibit weed germination, but also increase soil fertility and provide mineral elements for plants [4–6]. In addition, it also could increase the biodiversity of soil microecosystem [7].

Soil microorganism played important roles in nutrient cycling and structure maintain in the agro-ecosystem [8, 9]. Soil microbial diversity was involved in the circulation of matter and energy in the soil ecosystem [10]. The changes of soil physicochemical properties during mulching process can drive the changes of the microbial community [11], which affect the microbial community structure and metabolic function [12, 13]. Soil microbial diversity and biomass were sensitive to the changes in the soil physicochemical properties and managements, and were useful for predicting the changes in the soil functions [14–17]. Therefore, investigating the diversity and composition of microbial communities in soils under mulching would be conducive to take effective measures to improve the fertility and productivity of soils and ensure the sustainable development of soil ecosystems.

Tea plant (*Camellia sinensis L.*), an evergreen leafy plant, prefers acidic soil according to the genetic characteristics, thus, the soil environment of tea plantation is different from that of other crops. Several studies reported that mulching could increase the richness and diversity of microbial community in other crop soils [18, 19]. However, to our knowledge, the information about soil microbial diversity and composition in tea plantation with inorganic or organic mulching was limited. In the present study, we performed one field trial to research the bacterial and fungal diversity, community composition and functional annotation of soils under two different materials (inorganic, polyethylene film; organic, peanut hull) on two depths of soils (0-20 cm depth; 20-40 cm depth). According to Linear discriminant analysis Effect Size (LEfSe) tool, soil bacterial and fungal communities with significant differences under different materials were identified. According to the spearman correlation, the relationships between soil bacterial, fungal community structure and soil property were discussed. This study not only compared the microbial effects of different materials, but also provided theoretical support for the rational application of different mulching materials in tea plantation. The results are of great significance not only for improving soil quality, but also for improving fertilizer efficiency.

## Results

### The diversity of soil bacterial and fungal community

**Fig. 1:**
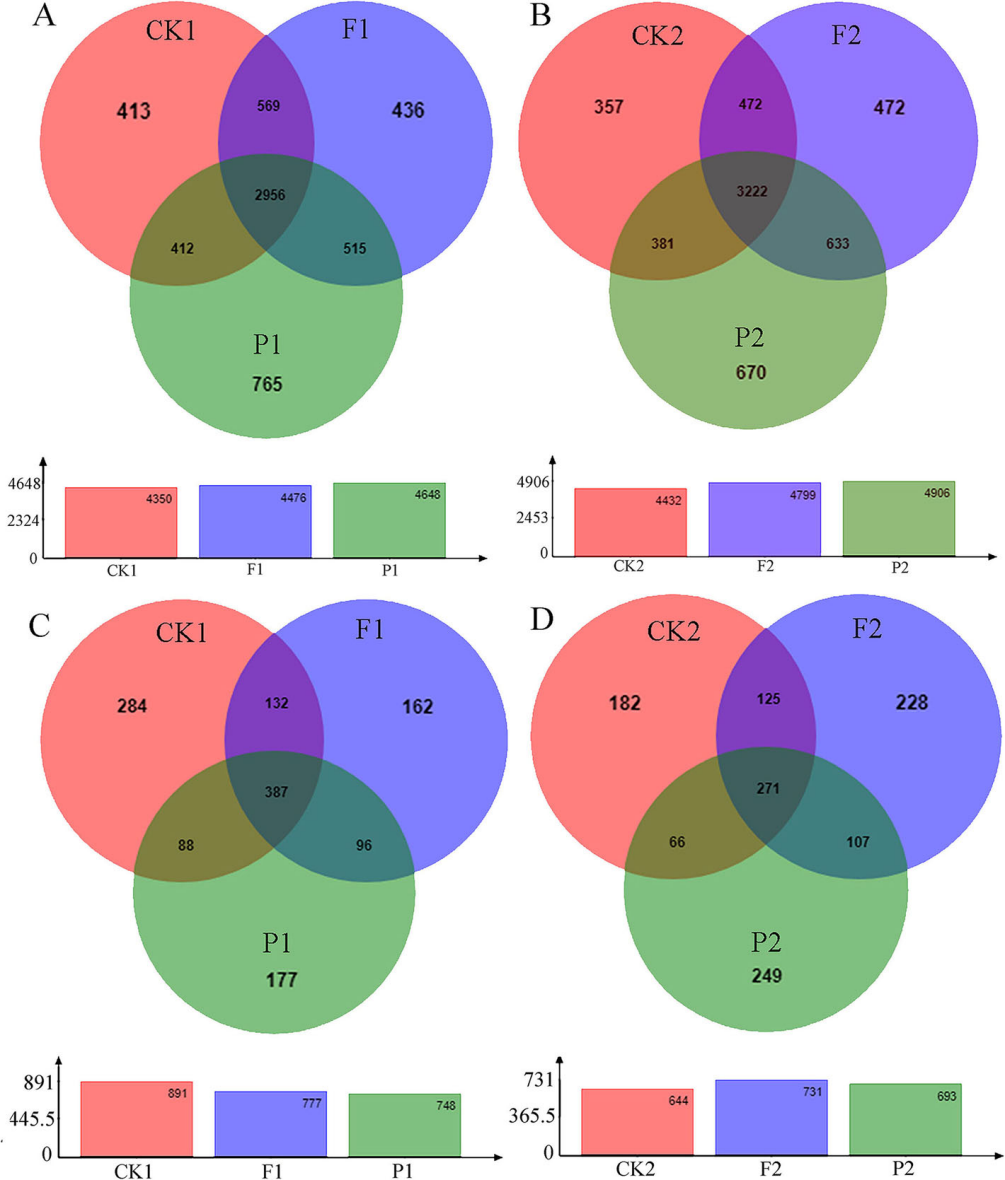
The Venn diagram of microbial communities in soils under different mulching patterns. **a** The number of bacterial OTUs in 0–20 cm depth soils under different mulching patterns. **b** The number of bacterial OTUs in 20–40 cm depth soils under different mulching patterns. **c** The number of fungal OTUs in 0–20 cm depth soils under different mulching patterns. **d** The number of fungal OTUs in 20–40 cm depth soils under different mulching patterns. CK1: bare soil control, 0-20 cm soil; CK2: bare soil control, 20-40 cm soil; F1: mulching with polyethylene film, 0-20 cm soil; F2: mulching with polyethylene film, 20-40 cm soil; P1: mulching with peanut hull, 0-20 cm soil; P2: mulching with peanut hull, 20-40 cm soil

To ensure the quality of sequencing of all samples, we optimized the original sequences using the quality filter and chimera check. A total of 576,090 high-quality reads of bacteria and 886,572 high-quality reads of fungi were remained in the dataset with the average length of 418 bp and 234 bp, respectively. Rarefaction curve analysis showed that each curve was close to flat finally, indicating that the sample size of the sequencing was sufficient and the sequencing data were reasonable and acceptable (Additional file 1: Figure S1). Through clustering operations, the optimized sequences were classified into operational taxonomic units (OTUs) according to their similarity. With a 3% dissimilarity threshold, the sequences were classified into 6809 and 1684 OTUs in bacterial and fungal communities using the Ribosomal Database Project (RDP) classifier. In bacterial communities, the Venn diagram showed that the numbers of OTUs in P1 and P2 were more than that in F1 and F2 (Fig. 1a-b), but in fungal communities, the numbers of OTUs were not significant differences in three treatments (Fig. 1c-d).

To quantify the diversity and richness of microbial community of soils among three treatments, we calculated the indices of alpha diversity such as Chao1, ACE, Simpson and Shannon within a single microbial ecosystem (Table 1). The coverage indexes from 18 soil samples were greater than 0.96, indicating that the sequencing capacity was acceptable. Variance analysis showed that the different mulching materials and different soil depths affected the bacterial and fungal communities. In 0–20 cm soil, the richness of P1 was slightly higher than that in CK1 and F1 in bacterial communities, while there was no significant difference. However, in fungal communities, the richness of P1 was significantly lower than that in CK1 and F1. In 20–40 cm soil, the richness of P2 was significantly higher than that in CK2 and F2 in bacterial communities. Thus, peanut hull mulching greatly affected the diversity of fungal communities in 0-20 cm soil and bacterial communities in 20-40 cm soils.

**Table 1:** The diversity indices of soil bacterial and fungal communities

Depth	Sample	Diversity index		Richness estimator		Coverage (%)
		Shannon	Simpson (%)	Ace	Chao1	
0–20 cm						
bactria	CK1	6.45 ± 0.41a	0.66 ± 0.45a	3781.83 ± 432.04 a	3744.08 ± 398.80a	97.14
	F1	6.60 ± 0.07a	0.45 ± 0.04a	3904.53 ± 198.55a	3909.83 ± 198.06a	97.02
	P1	6.65 ± 0.05a	0.61 ± 0.16a	4201.73 ± 116.90a	4173.82 ± 135.26a	96.77
fungi	CK1	4.07 ± 0.37a	3.99 ± 1.63b	593.23 ± 13.98 a	582.66 ± 23.45a	99.80
	F1	3.70 ± 0.49a	6.07 ± 2.87ab	537.89 ± 13.42b	553.00 ± 13.47ab	99.80
	P1	3.48 ± 0.40a	8.92 ± 2.33a	494.43 ± 35.60b	514.27 ± 30.90b	99.84
20–40 cm						
bactria	CK2	6.75 ± 0.05b	0.34 ± 0.02a	4030.42 ± 128.36c	4034.75 ± 152.51b	96.96
	F2	6.80 ± 0.04b	0.34 ± 0.02a	4236.75 ± 54.29b	4163.55 ± 75.29b	96.81
	P2	6.90 ± 0.06a	0.31 ± 0.03a	4576.91 ± 305.98a	4545.10 ± 331.09a	96.48
fungi	CK2	3.92 ± 0.06a	4.80 ± 0.91a	375.66 ± 71.62a	385.11 ± 63.80a	99.94
	F2	4.21 ± 0.28a	3.57 ± 1.33a	411.62 ± 84.00a	412.16 ± 85.67a	99.92
	P2	3.02 ± 1.14a	20.94 ± 0.21a	455.92 ± 89.48a	461.94 ± 84.05a	99.87

The mean value ± standard deviation (*n* = 3). Values with the same letter are not significantly different (*p* < 0.05)

**Fig. 2:**
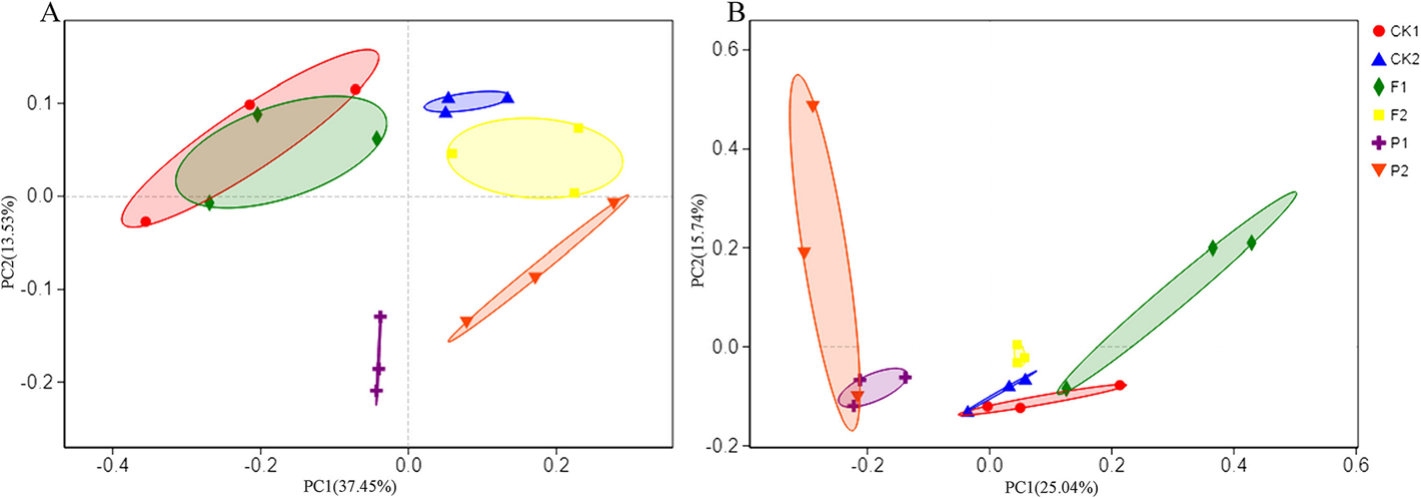
The principal coordinate analysis (PCoA) based on Bray–Curtis distance in microbial communities in soils under different mulching patterns. **a** The distribution of bacterial communities in soils under different mulching patterns. **b** The distribution of fungal communities in soils with different mulching patterns. CK1: bare soil control, 0-20 cm soil; CK2: bare soil control, 20-40 cm soil; F1: mulching with polyethylene film, 0-20 cm soil; F2: mulching with polyethylene film, 20-40 cm soil; P1: mulching with peanut hull, 0-20 cm soil; P2: mulching with peanut hull, 20-40 cm soil

To dig into the effects of different mulching materials on the distribution of soil microbial communities, we made the principal co-ordinates analysis (PCoA) based on the bray–curtis distance. The first principal coordination axis accounted for 37.45 and 25.04% in bacterial and fungal communities, respectively. The second principal coordination axis explained 13.53 and 15.74% in bacterial and fungal communities, respectively. In bacterial communities, there were significant differences between 0 and 20 cm soils and 20–40 cm soils: CK2, F2 and P2 were on the positive side of the x-axis, and CK1, F1and P1 were on the negative side. Furthermore, there were significant differences between the mulching plastic film and peanut shell on the topsoil: P1 and P2 were on the positive side of the y-axis, and CK1, CK2, F1 and F2 were on the negative side (Fig. 2a). Therefore, the distribution of bacterial communities was mainly affected by soil depths and mulching materials, especially organic mulches. In fungal communities, P1 and P2 clustered more closely together on the positive side of x-axis and CK1, CK2, F1 and F2 were on the negative side, indicating that the organic mulch could affect the distribution of fungal communities in soils (Fig. 2b).

### The composition of soil bacterial and fungal community

**Fig. 3:**
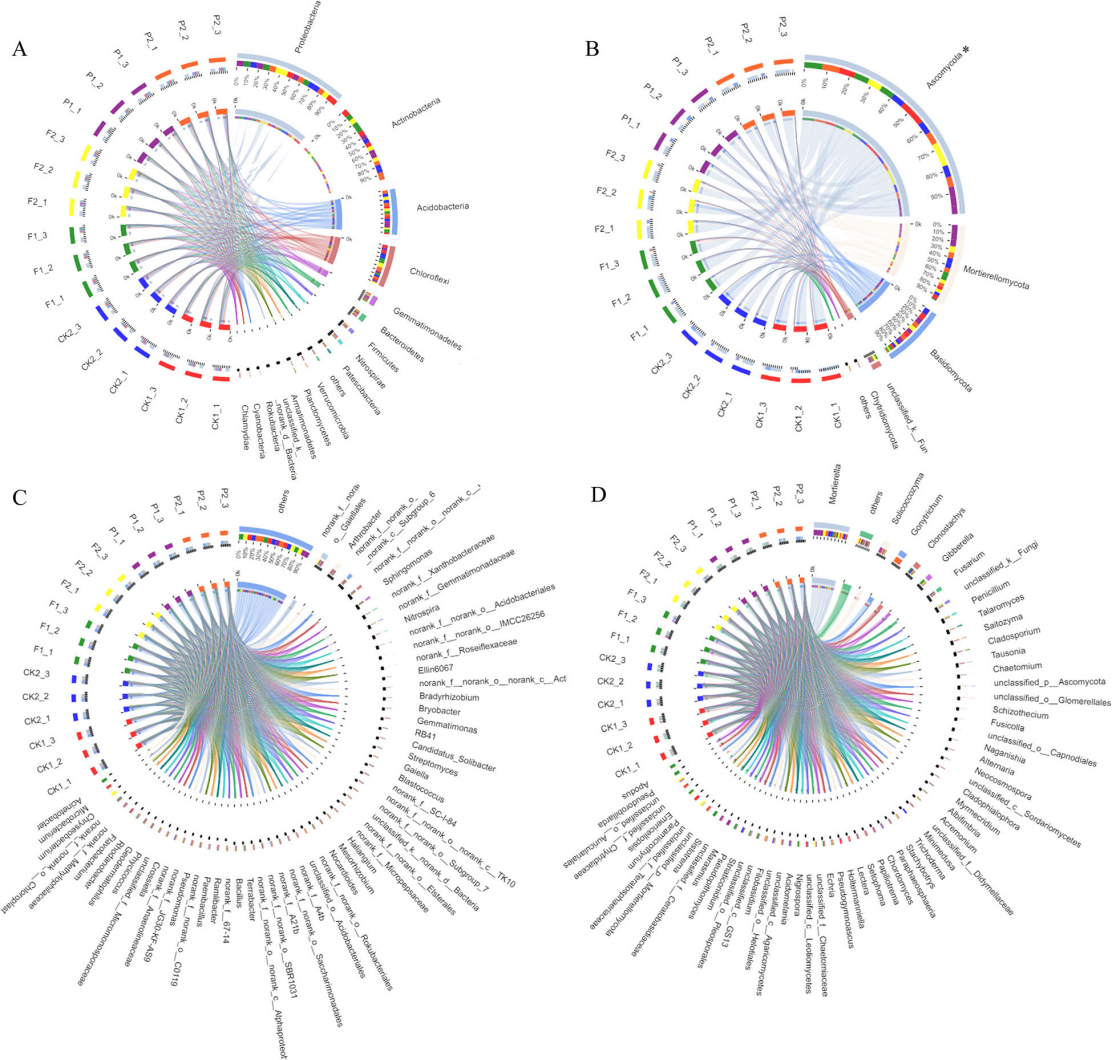
The composition of microbial community in soils under different mulching patterns. **a** The composition of bacterial community at phylum level. **b** The composition of fungal community at phylum level. **c** The composition of bacterial community at genus level. **d** The composition of fungal community at genus level. The data were visualized by Circos. The width of the bars from each phylum indicated the relative abundance of the phylum

**Fig. 4:**
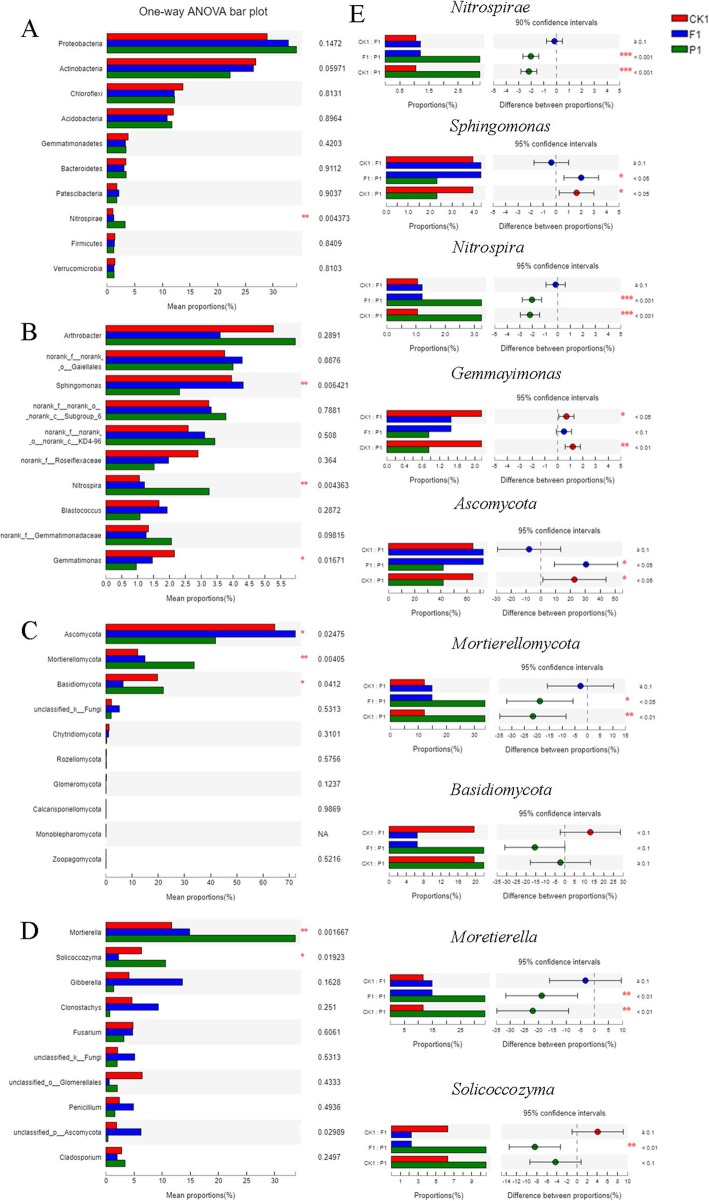
The relative abundance of main microbial community in soils under different mulching patterns. **a** The relative abundance of top10 bacterial community at phylum level. **b** The relative abundance of top10 bacterial community at genus level. **c** The relative abundance of top10 fungal community at phylum level. **d** The relative abundance of top10 fungal community at genus level. **e** The microbial communities with significant differences in soils under different mulching patterns

To dissect the composition of soil microbial community, we aligned the top30 OTUs with the Silva 119 and Unite database at the phylum and genus levels. The dominant bacterial phyla were Proteobacteria, Actinobacteria, Acidobacteria and Chloroflexi (Fig. 3a), and the dominant fungal phyla were Ascomycota, Mortierellomycota and Basidiomycota (Fig. 3b). The dominant bacterial genus were *norank_o__Gaiellales, Arthrobacter, norank_c__Subgroup_6, norank_c__KD4–96* and *Sphingomonas* (Fig. 3c), and the dominant fungal genus were *Mortierella, Solicoccozyma, Fusarium and Clonostachys* (Fig. 3d). In addition, there were significant differences in bacterial and fungal communities in soils under polyethylene film and peanut hull mulching (Fig. 4). At the phylum level, Nitrospirae presented significant differences in bacterial communities, and Mortierellomycota, Ascomycota and Basidiomycota presented significant differences in fungal communities. Therein, the relative abundance of Nitrospirae in P1 (3.24%) was significantly higher than that in F1 (1.21%) and CK1 (1.05%) of bacterial communities, and the relative abundances of Mortierellomycota (33.72%) and Basidiomycota (21.93%) in P1 was significantly higher than that in F1 and CK1of fungal communities. At the genus level, *Sphingomonas*, *Nitrospira* and *Gemmayimonas* presented significant differences in bacterial communities, *Moretierella* and *solicoccozyma* presented significant differences in fungal communities. Therein, the relative abundances of *Nitrospira* (3.24%) was significantly higher than that in F1 (1.21%) and CK1 (1.05%) of bacterial communities, and the relative abundances of *Moretierella* (33.66%) and *solicoccozyma* (10.60%) in P1 was significantly higher than that in F1 and CK1 of fungal communities.

To identify the bacterial and fungal communities with significant differences in soils among three treatments, we further analyzed microbial community in 0-20 cm soil from phylum to genus level using LEfSe tool (Additional file 2: Figure S2; Additional file 3: Figure S3). Eight groups of bacteria and two groups of fungi in P1 were significantly enriched, of which, bacteria included Gammaproteobacteria (class and the genus *IS_44*), Xanthobacteraceae (family), Parcubacteria (class)*,* Amb_16S_1323 (the family and its genus), Candidatus_Magasanikbacteria (the order to genus), WS6_Dojkabacteria (the class to genus), UA_11 (the order to genus) and *Paludibaculum* (genus), and fungi included Piskurozymaceae (family and the genus *Solicoccozyma*) and Leucosporidiales (the order to genus). Three groups of bacteria and two groups of fungi in F1 were significantly enriched, of which, bacteria included Moraxellaceae (family), Solirubrobacteraceae (family) and Caulobacteraceae (family), and fungi included *Acremonium* (genus) and *Emericellopsis* (genus). The results showed that bacterial and fungal communities with significant differences in soils under different mulching were the noteworthy microbial communities in tea plantation.

### Relationships between the preponderant phyla of microbial communities and the characteristics of soils

Firstly, to evaluate the physicochemical characteristics of soils under different mulching patterns, we analyzed the contents of nutrients in 0–20 cm and 20–40 cm depths soils (Table 2). In the topsoil, the content of TN in P1 was observably higher than that in CK1 and F1, and the soil moisture in P1 was significantly higher than that in CK1 and F1. While the contents of OM, AN and AP were slightly higher than those in CK1 and F1, but there was no significant difference. The pH value was F1 > P1 > CK1. In 20-40 cm soil, the contents of TN and TP in CK2 were observably higher than that in F2 and P2, and the soil moisture in P2 was significantly higher than that in CK2 and F2.

**Table 2:** Soil physicochemical characteristics in different mulch treatments

	pH	Moisture (%)	OM (g/kg)	TN (g/kg)	TP (g/kg)	TK (g/kg)	AN (mg/kg)	AP (mg/kg)	AK (mg/kg)
CK1	6.82 ± 0.17a	9.4 ± 0.40a	11.92 ± 1.50a	0.24 ± 0.01c	1.70 ± 0.48a	17.34 ± 1.09a	91.18 ± 15.14a	16.60 ± 2.29a	87.56 ± 7.75a
F1	6.99 ± 0.32a	10.2 ± 0.25a	12.48 ± 2.61a	0.36 ± 0.03b	1.40 ± 0.14a	21.73 ± 9.52a	98.45 ± 6.67a	12.36 ± 6.97a	83.60 ± 2.15a
P1	6.86 ± 0.10a	12.3 ± 0.75b	15.14 ± 3.29a	0.55 ± 0.03a	1.33 ± 0.08a	19.25 ± 12.42a	109.08 ± 5.69a	15.78 ± 0.36a	86.19 ± 5.08a
*F*-value	0.479	23.963	1.339	162.641	1.325	0.177	2.383	7.170	1.746
*P*-value	0.641	0.001	0.331	0.000	0.334	0.842	0.173	0.026	0.253
CK2	7.16 ± 0.11a	10.0 ± 0.31a	11.40 ± 1.24a	0.33 ± 0.03a	1.95 ± 0.18a	19.22 ± 11.86a	95.74 ± 5.25a	16.42 ± 2.56a	79.81 ± 1.58a
F2	7.16 ± 0.15a	10.3 ± 0.36a	10.47 ± 1.33a	0.23 ± 0.04b	1.46 ± 0.30b	21.67 ± 9.84a	95.31 ± 9.75a	12.40 ± 7.69a	77.57 ± 2.59a
P2	7.07 ± 0.17a	12.8 ± 1.33b	10.48 ± 1.83a	0.23 ± 0.01b	1.39 ± 0.22b	18.30 ± 4.40a	96.49 ± 4.15a	14.45 ± 5.54a	78.95 ± 10.90a
*F*-value	0.363	10.237	0.386	14.606	5.032	0.106	0.023	0.146	0.691
*P*-value	0.710	0.012	0.695	0.005	0.052	0.901	0.977	0.867	0.537

The mean value ± standard deviation (*n* = 3). Values with the same letter are not significantly different (*p* < 0.05)

**Fig. 5:**
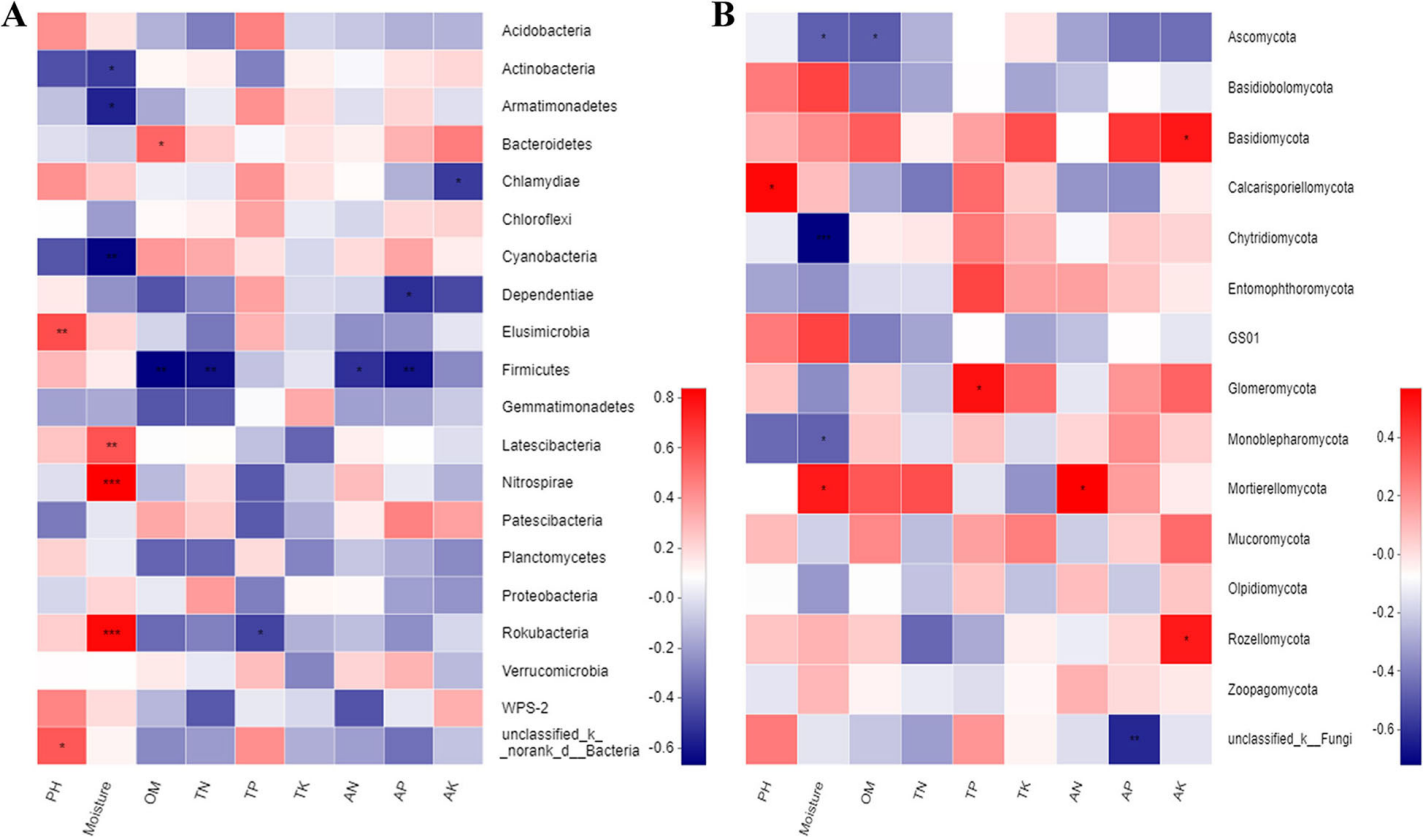
The spearman correlation heatmap between soil physicochemical characteristics and microbial communities in soils under different mulching patterns. **a** The relationships between soil physicochemical characteristics and bacterial communities. **b** The relationships between soil physicochemical characteristics and fungal communities

Secondly, to explore the influences of soil environmental factors on soil bacterial and fungal communities, we analyzed the relationships between microbial preponderant phyla and main soil properties using spearman correlation heatmap (Fig. 5). In bacterial communities, the soil pH was positively related with the abundance of Acidobacteria and Elusimicrobia*.* The soil moisture was positively related with the abundances of Nitrospirae and Rokubacteria, while it was negatively related with Cyanobacteria and Actinobacteria. The soil OM content was positively related with Bacteroidetes, while it was negatively related with the abundances of Firmicutes. The abundances of Firmicutes was positively interrelated with the contents of soil OM, TN, AN and AP. In fungal communities, the soil moisture was positively correlated to the abundances of Mortierellomycota, while it was negatively correlated to Chytridiomycota and Ascomycota. The soil OM was negatively correlated to Ascomycota. The AP and AK contents were positively correlated to Basidiomycota, while they were negatively correlated to the abundances of Ascomycota. The soil AN content was positively correlated to Mortierellomycota. The results showed that soil physicochemical characteristics had great influences on the structure of microbial communities.

### Functional prediction of soil microbial communities

**Fig. 6:**
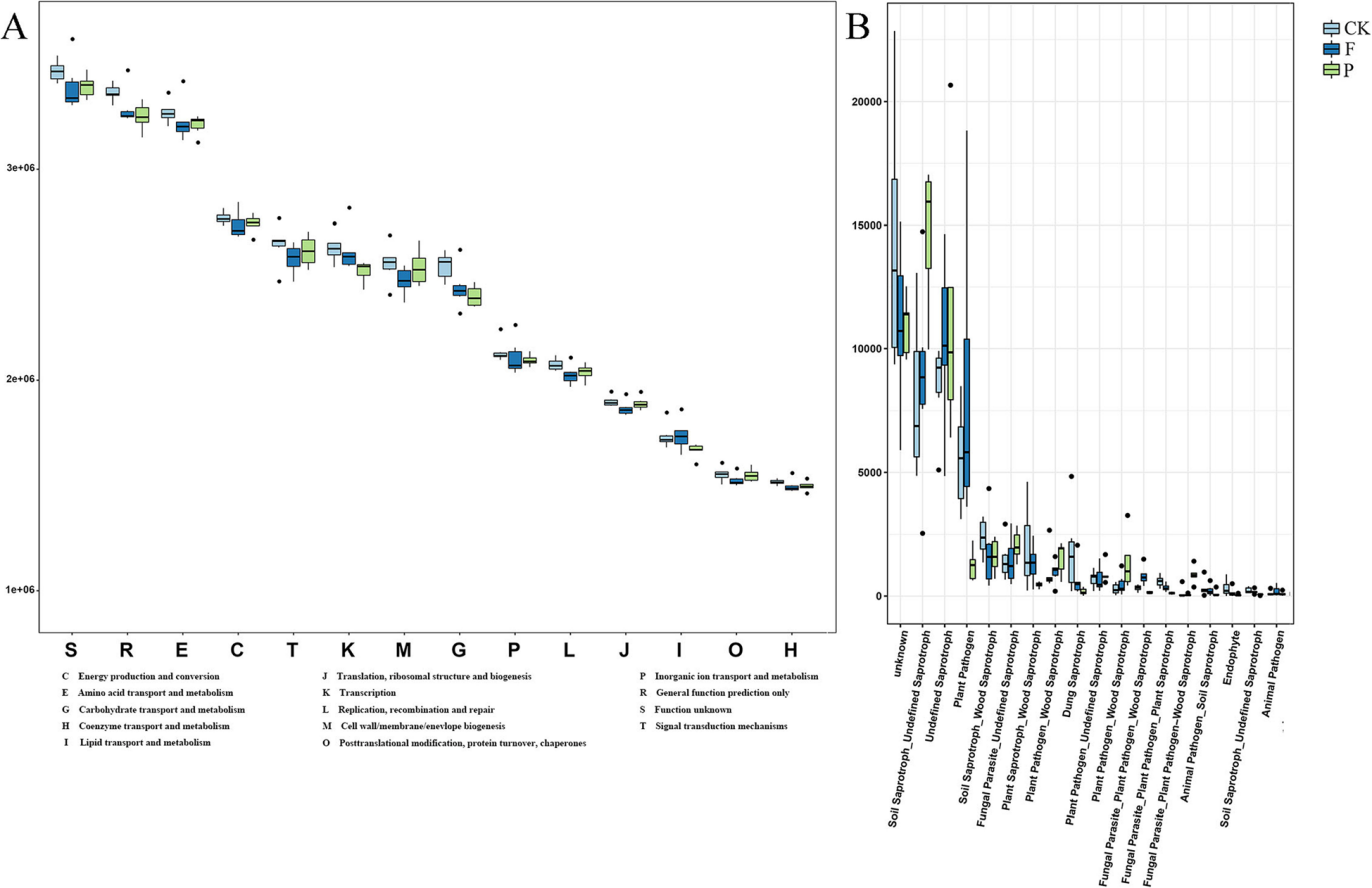
The microbial communities functional features in soil under different mulches. **a** Bacterial community functional features. **b** Fungal community functional features. CK: bare soil control; F: mulching with polyethylene film; P: mulching with peanut hull

To better study the micro-ecological functions of the soil microorganisms in tea plantation under organic and inorganic mulching patterns, we analyzed the bacterial communities in the Cluster of Orthologous Groups (COG) database using PICRUSt (Fig. 6a). The results showed that the bacterial functional features were related to different treatments. It could be observed the metabolic functions were enriched in soil samples and the bacterial metabolism tended to be vigorous. These functional features included: General function prediction; Amino acid transport and metabolism; Energy production and conversion; Signal transduction mechanisms; Transcription; Cell wall/membrane/envelope biogenesis; Carbohydrate transport and metabolism; Inorganic ion transport and metabolism; Replication, recombination and repair. Therein, the relative abundances of amino acid transport and metabolism, Energy production and conversion and signal transduction mechanisms in soils under peanut hull mulching were higher than that in soils under polyethylene film mulching.

In addition, we assigned fungal OTUs to specific nutritional groups, and then subdivided them into specific ecological associations (Fig. 6b). FunGuild was a set of designations, which established fungal classification tool with rigorously defined and cited nutrition group assignments. Three ecological functions, including undefined saprotrophs, plant pathogens and soils aprotrophs, were significantly enriched. The relative abundances of undefined saprotroph and soil saprotroph in soils with peanut hull mulching were higher than other under ecological functions.

## Discussion

The mulching patterns on soils with different materials were widely adopted in agricultural cultivation for a long time. The general effect of mulching on soils has also been fully recognized. However, in terms of the influence of different mulching materials on the composition and function of microorganisms in soils, the depth of understanding is far from enough. In the study, in order to reveal the effects of mulching on microbial communities, we analyzed the bacterial and fungal diversity and community composition in tea plantation under polyethylene film or peanut hull using 16S rRNA and ITS rDNA, respectively. The results indicated that mulching could change soil properties and impact soil microbial diversity and community structure, and improve ecosystem functions of tea plantation.

The diversity of soil microbial community was critical to the integrity, stability and sustainability of soil ecosystems [20]. It was affected by different agricultural management, such as different mulching patterns. The mulches of crop residues and straw on soils in the orchard had higher enzymatic activity, microbial biomass, showing that this management to soils under organic mulching was an effective and sustainable agricultural technology. As it produced the increase of the different fractions of organic carbon and microbial activity, it will translate into rapid improvement of soil quality [21]. In an urban land cover experiment, the diversity of microbial community on bark-mulched soils was higher than that of gravel-mulched and unmanaged fields, showing that organic mulches could provide nutrient-rich substrates for fueling microbial richness in soils [22]. However, little research had focused on the patterns of organic and inorganic mulching on soil in tea plantations could affect soil microbial diversity. In the present study, peanut hull mulching increased the diversity of fungal communities in 0–20 cm soil and the richness of bacterial communities in 20–40 cm soil (Table 1). The results indicated that the effect of mulching materials on the diversity of the bacteria was different from that of the fungi. High nutrient contents of peanut hull transported organic matter to soil system, which ultimately caused the quickly reproduction of soil microbes and increased the diversity of microbial communities. On the other hands, we analyzed the distribution of microbial communities in tea plantation soils under different mulching materials. The principal coordinates analysis of microbial communities further revealed the clear separation between organic and inorganic mulching, indicating that there were significant differences in the structures of bacterial and fungal communities in soils among organic and inorganic mulching patterns (Fig. 2). Thus, the organic and inorganic mulching on tea plantation soils constructed different relatively independent micro-ecological system. Compared with film mulching and no mulching, the organic mulching in tea plantation could obtain more abundant and diverse micro-ecological environment.

The composition structure of soil microbial communities were affected by different mulching patterns. In the present study, the results showed that the dominant bacterial phyla in tea plantation were Proteobacteria, Actinobacteria, Acidobacteria and Chloroflexi (Fig. 3a). Proteobacteria are the important phyla in the microbial community. They could be capable to exploit labile carbon sources and had higher relative abundances in nutrient-rich environments [23, 24]. Acidobacteria are the common eosinophil phyla in soils. They had the ability to degrade the cellulose and lignin of plant residues and played an important role in the carbon cycle [25, 26]. High abundances of Proteobacteria and Acidobacteria can enhance cycling of essential nutrients, which can improve soil fertility and sustainable utilization [27, 28]. Our results showed that the relative abundances of Proteobacteria and Acidobacteria in soil under peanut-hull mulching were higher than that with polyethylene-film mulching, indicating that peanut hull as crop residues could provide rich nutrients for Proteobacteria and the inside substance could be used by Acidobacteria (Fig. 4a)*.* Actinobacteria are mostly saprophytes in the soil. They were functionally diverse and contributed to the decomposition of organic matter, which they had ability to break down complex substrates to give them a competitive advantage over other bacteria [29, 30]. Chloroflexi are the important phyla with green pigment. They exhibited oligotrophic lifestyles and grew at a lower rate under conditions of higher nutrient utilization [31]. Our results showed that the relative abundance of Actinobacteria and Chloroflexi in soils with polyethylene film mulching was higher than that in soils with peanut hull mulching, indicating that the application of polyethylene film created a relatively nutrient-poor environment than peanut hull (Fig. 4a). In addition, we further digged into the significant accumulation of *Sphingomonas* and *Nitrospira* at on bacterial genus level (Fig. 4e). The genus *Sphingomonas* had extensive metabolic ability to degrade many aromatic compounds and the certain species of this genus could synthesize valuable extracellular biopolymers [32]. Therefore, the accumulation of *Sphingomonas* in soils might be caused by mulching, which might affect the growth of tea plant by affecting the root secretions. The genus *Nitrospira* were involved in nitrification process in many natural environments [33], and they played important roles in biogeochemical nitrogen cycle in soils [34]. The bacteria in the genus *Nitrospira* promoted the conversion of nitrite to nitrate by nitrification in soils [35, 36]. The soils covered by peanut hull had the potential advantage of exogenous nitrate intervention, which significantly increased the competitive advantage of *Nitrospira*. High relative abundance of *Nitrospira* in soils under peanut-hull mulching indicated that the organic mulching could enrich bacteria to involve in the nitrogen cycle and promote the biochemical cycle in tea plantations.

On the other hands, in the present study, the dominant fungal phyla were Ascomycota, Mortierellomycota and Basidiomycota (Fig. 3b), and the dominant fungal genus were *Mortierella, Solicoccozyma, Fusarium and Clonostachys* (Fig. 3d). Ascomycota were the main fungal decomposer in many soil ecosystems [37–39]. As biotrophs, they played the biggest role in recycling plant residues, and they might form symbioses with mycorrhizae or endophytes [40]. Basidiomycota played an important role in degrading lignin under anaerobic conditions [41]. Our results showed that the relative abundance of Basidiomycota in soils under peanut hull mulching was significantly higher than that in soils under polyethylene-film mulching, indicating that peanut hull could provide anaerobic and high lignin content for the growth of Basidiomycota*,* and it could make Basidiomycota attend the conversion of lignin under anaerobic conditions (Fig. 4e). At fungal genus level, *Mortierella* were known as saprobic and ubiquitous. Numbers of studies proved that they had ability to solubilize phosphorus, and were related to increase crop yields and establish symbiosis with plants [42, 43]. In our study, *Mortierella* showed significant accumulation in soils under peanut hull mulching, indicating that good quality soils in tea plantation were primarily related with high relative abundance of *Mortierella*. In addition, the genus *Fusarium* was important crop pathogens in the world. The disease caused by *Fusarium* could infect different host organs, including plant seeds and roots [44]. They were capable of colonising root surfaces and produced some plant irritant, but they also produced some secondary metabolite toxin during growth and metabolism, which could cause negative plant-soil feedback [45, 46]. Our study showed *Fusarium* was the dominant fungal genus in tea plantation soil, but the relative abundance of *Fusarium* in soils under film mulching was higher than that in soils under peanut hull mulching. Therefore, covering with peanut shell might reduce the accumulation of soil borne pathogens and the occurrence of soil disease in tea plantation.

Agricultural practices in tea plantations could alter soil environmental factors [37]. Soil environmental factors, in turn, further affected the composition of microbial communities [47, 48]. Previous studies in an apple orchard and maize system reported that the application of mulch enhanced water-holding capacity and soil nutrients [49, 50]. In the present study of tea plantation, soil moisture and soil TN contents with peanut-hull mulching were significantly higher than those in soils with plastic-film mulching on the topsoil (Table 2), indicating that the organic mulch had better adsorption effect on soil moisture and rainfall, and it could also provide nitrogen source for soil microorganisms. Spearman correlation heatmap analysis showed that the dominant bacterial and fungal phyla were significantly correlated with soil properties (Fig. 5). Soil moisture and soil total nitrogen content were the main physicochemical factors that affect the structure of bacterial community. Meanwhile, the soil moisture and available nitrogen contents were the main physicochemical factors that affect the structure of fungal community in tea plantation. Mulch was beneficial to adjust a microbial community with stronger interaction and could enhance the functional ability of the soil microbes. Therefore, different mulching methods altered the soil microbial communities of tea plantation by regulating soil properties.

## Conclusions

Our study highlighted the influence of different mulches on soil microbial diversity and community structure in tea plantation. It evaluated the potential value of organic mulching for tea garden management from two aspects of microbial structure and ecological function. The results showed that organic mulching improved the physicochemical conditions of tea garden soils and induced great changes of soil bacterial and fungal communities, and also improved the soil micro ecological environment. Compared with film mulching and no mulching, the organic mulching in tea plantation could obtain more abundant and diverse micro-ecological environment. So, it is a better choice for tea plantation management. In addition, it is necessary to strengthen the research on the changes of rhizosphere microorganisms under different mulching treatments in the future. At the same time, because the metabolism of soil microorganism is related to the production performance of tea plant, it is also necessary to further research the effect of soil microorganism on the growth and development of new shoots of tea plant.

## Methods

### Field experiment and soil sampling

The field experiment was conducted from May 2019 at Rizhao in Shandong Province of China (35°04′N, 118°25′E). This region has atypical subtropical monsoon climate with an average annual precipitation of 1048 mm and an average temperature of 12.7 °C. The soil of the experimental field is brown soil, which is cleared and hoed manually before the experiment. The major soil parameters were as follows: pH 6.8, organic matter (OM, 11.92 g/kg), available nitrogen (AN, 91.18 mg/kg), available phosphorus (AP, 16.60 mg/kg) and available potassium (AK, 87.56 mg/kg).

The treatments were: (1) CK1: bare soil control, 0-20 cm soil; (2) CK2: bare soil control, 20-40 cm soil; (3) F1: mulching with polyethylene film, 0-20 cm soil; (4) F2: mulching with polyethylene film, 20-40 cm soil; (5) P1: mulching with peanut hull, 0-20 cm soil; and (6) P2: mulching with peanut hull, 20-40 cm soil. Each plot was 90 m^2^ under unified management and each treatment had three randomly replicate plots. The tea tree variety was ‘Zhongcha108’ with a row spacing of 1.5 m, and plant spacing of 0.33 m. The polyethylene film was black with a thickness of 0.004 mm, and was compacted with soil around the cover. The peanut hull was mulched in the soil surface only with a thickness of 10 cm. Prior to coring, mulch was removed from sampling area to prevent contamination of cores with surface organic matter. The soil sampling in every depth was homogenized by mixing together and passing through a 2-mm sieve for discarding mixed above-ground materials (plant residues and stones). Each soil sample with 3 replications was further divided into two parts: one part was frozen quickly in liquid nitrogen, and stored at − 80°Cuntil the microbial communities were analyzed; the other part was dried naturally in room and then analyzed for pH, available nitrogen (AN), available phosphorus (AP), available potassium (AK), organic matter (OM), total nitrogen (TN), total phosphorus (TP), total potassium (TK).

### DNA extraction and PCR amplification

According to the manufacturer’s protocols, microbial DNA from 18 samples was extracted from 18 samples in soils and the E.Z.N.A.® Soil DNA Kit (Omega Bio-tek, Norcross, GA, U.S.) was used. The final DNA concentration and purified concentration and purification were determined by NanoDrop 2000 ultraviolet-visible spectrophotometer, and the DNA quality was checked by 1% agarose gel electrophoresis. The concentration and purification of final DNA were determined by NanoDrop 2000 UV-vis spectrophotometer (Thermo Scientific, Wilmington, USA), and the DNA quality was checked by 1% agarose gel electrophoresis. The DNA samples were individually amplified in V3-V4 hypervariable regions by PCR using primers 338F (5′-ACTCCTACGGGAGGCAGCAG − 3′) and 806R (5′-GGACTACHVGGGTWTCTAAT − 3′) for 16S rDNA in bacteria, and primers ITS1F (5′-CTTGGTCATTTAGAGGAAGTAA − 3′) and ITS2R (5′-GCTGCGTTCTTCATCGATGC-3′) for ITS in fungi. The procedure used in the PCR reaction had been described in previous study [51]. In short, the PCR reaction was repeated 3 times, and it should go through the procedures of denaturation, annealing, and extension. Then, the resulting PCR product was extracted from a 2% agarose gel and further purified and quantified using the AxyPrep DNA gel extraction kit (Axygen Biosciences, Union City, California, USA) and QuantiFluor™ -ST (according to the manufacturer’s protocol US Promega), respectively.

### Illumina sequencing and processing of sequencing data

The purified amplicons were pooled on the Illumina MiSeq platform (Illumina, San Diego, USA) of equal molecular weight and paired-end sequencing (2 × 300) according to the standard protocol of MajorbioBio-Pharm Technology Co. Ltd. (Shanghai, China).

The original sequencing sequence was controlled using Trimmomatic software and merged by FLASH software. The specific criteria are consistent with previous study [51]. UPARSE (version 7.1; http://drive5.com/uparse/) was used to cut the similarity of the operational classification units (OTUs) to 97%. *http://drive5.com/uparse/*) uses a novel “greedy” algorithm that could perform chimera filtering and OTU clustering at the same time. Using the confidence threshold of 70%, the classification of each 16S rRNA gene sequence was analyzed against the Silva database (Release132; *http://www.arb-silva.de*) through the RDP classifier algorithm (*http://rdp.cme.msu.edu/*). Using the confidence threshold of 70%, the classification of each ITS sequence was analyzed against the Unite database (version 7.2; *http: //unite.ut.ee/index.php*) through the RDP classifier algorithm.

### Statistical analysis

The means and standard deviations of the data were calculated and statistically examined by ANOVA and Duncan’s multiple range tests by SPSS (SPSS, Inc., Chicago, IL, USA). Significant differences were considered as *p* < 0.05. Alpha diversity was applied to analyze microbial diversity, including the Chao1, Shannon, Ace, Simpson and rarefaction curve, which were calculated with Mothur software (version 1.30.1, *http://www.mothur.org/*). The principal co-ordinates analysis (PCoA) was applied to reduce the dimension of the original variables based on the Bray-Curtis distances using R2.1.3. The Circos graph was built using Circos-0.67-7 software (*http://circos.ca/*) [52]. Linear discriminant analysis (LDA) coupled with effect size measurements (LEfSe) analysis was applied to search for statistically different biomarkers between different treatments using LEfSe software (*http://huttenhower.sph.harvard.edu/galaxy/*). The relationship between soil microbial community structures and soil environment factors was analyzed by Spearman correlation heatmap using pheatmap package.

## Supplementary information

**Additional file 1: Figure S1.** The rarefaction curve of bacterial (A) and fungal (B) communities in soils under different mulching patterns.

**Additional file 2: Figure S2.** The cladogram of the phylogenetic distribution of bacterial communities in soils under different mulching patterns.

**Additional file 3: Figure S3.** The cladogram of the phylogenetic distribution of fungal communities in soils under different mulching patterns.

## Data Availability

The raw sequencing data have been deposited in NCBI Sequence Read Archive (SRA) under accession number PRJNA614599 for bacteria, and PRJNA615029 for fungi.

## Abbreviations

AN: Available nitrogen
AP: Available phosphorus
AK: Available potassium
COG: Cluster of Orthologous Groups
ITS: Internal transcribed spacer
LEfSe: Linear discriminant analysis Effect Size
OM: Organic matter
OTU: Operational Taxonomic Units
PCoA: Principal co-ordinates analysis
RDA: Redundancy analysis
RDP: Ribosomal Database Project
TK: Total potassium
TN: Total nitrogen
TP: Total phosphorus
