# Supplementary figures and images for: Organic mulching positively regulates the soil microbial communities and ecosystem functions in tea plantation
Source: BMC Microbiol. 2020 Apr 29;20:103. doi: 10.1186/s12866-020-01794-8 (PMC7191807; doi:10.1186/s12866-020-01794-8)

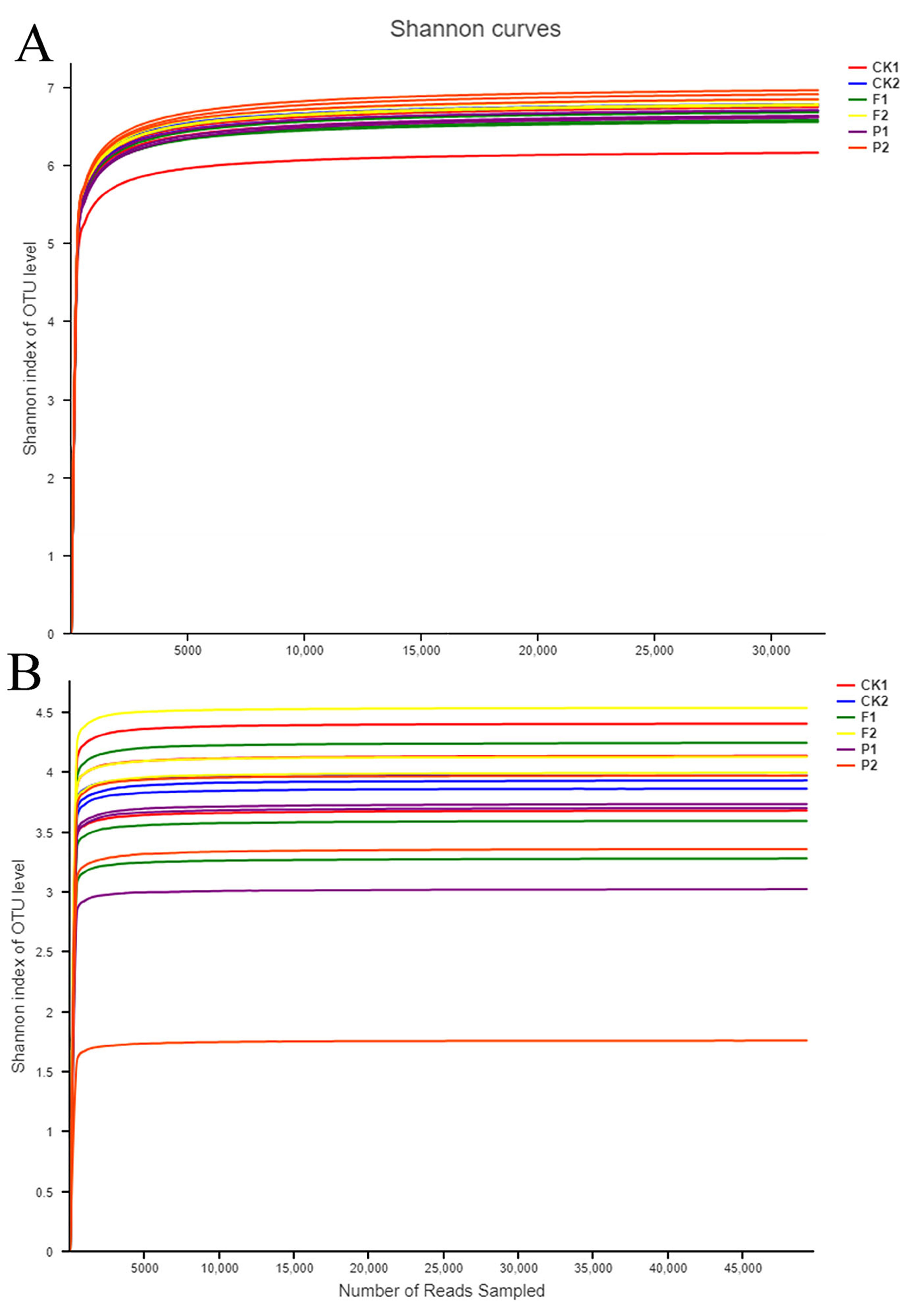

Supplement: Supplementary file 1 — Additional file 1: Figure S1. The rarefaction curve of bacterial (A) and fungal (B) communities in soils under different mulching patterns. [file 12866_2020_1794_MOESM1_ESM.jpg]

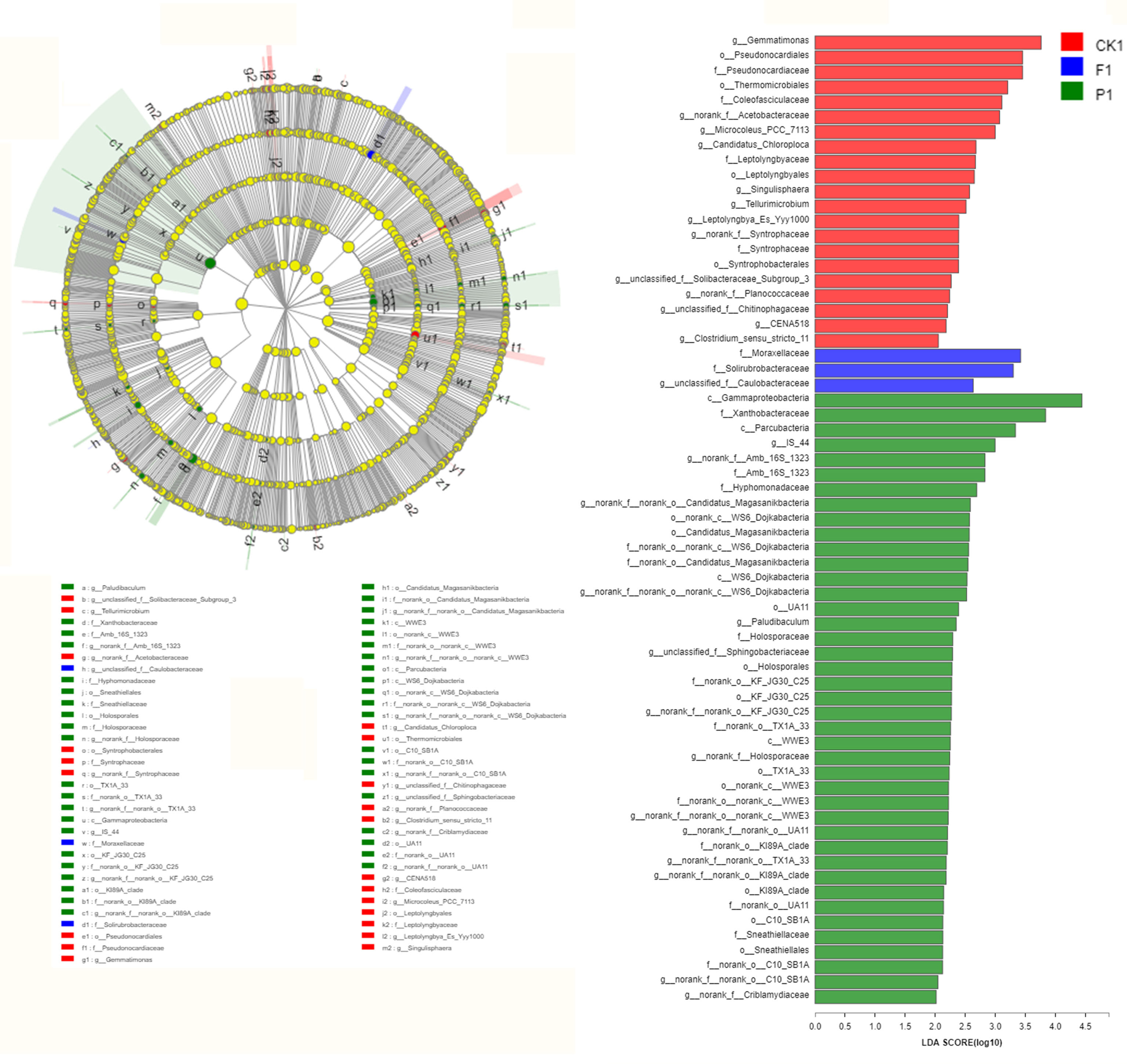

Supplement: Supplementary file 2 — Additional file 2: Figure S2. The cladogram of the phylogenetic distribution of bacterial communities in soils under different mulching patterns. [file 12866_2020_1794_MOESM2_ESM.jpg]

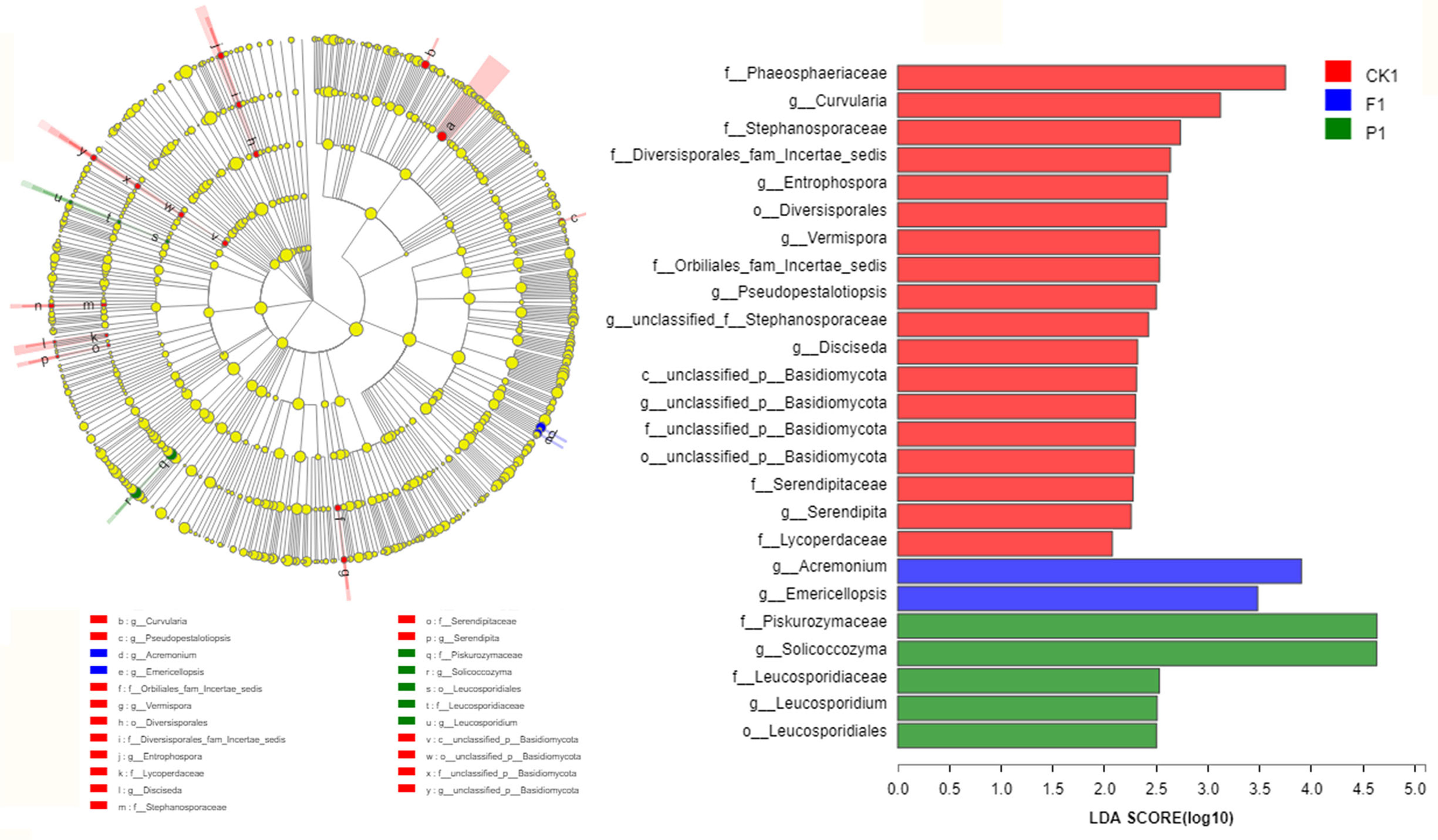

Supplement: Supplementary file 3 — Additional file 3: Figure S3. The cladogram of the phylogenetic distribution of fungal communities in soils under different mulching patterns. [file 12866_2020_1794_MOESM3_ESM.jpg]
